# Reports on the Hospitals of the United Kingdom

**Published:** 1912-03-09

**Authors:** Henry Burdett


### REPORTS ON THE Hospitals of the United Kingdom.

By SIR HENRY BURDETT, K.C.B., K.C.V.O.

### TWO CHILDREN'S HOSPITALS.

### LXIV.—The Children's Hospital, Sheffield.

This hospital was established in 1876, and has since been extended on three separate occasions—namely, in 1896, 1901, and 1906. It is remarkable from the fact that the present matron, Miss Pountney, who was trained at Brownlow Hill, Liverpool, became matron in 1881 and has held office ever since, carrying through successfully the extended organisation and many important changes which these enlargements have rendered necessary. After thirty years' continuous responsibility as matron Miss Pountney is active and more thoroughly interested in the work than many much younger women. She has the reputation—and it is evidently deserved—of being so thorough in her work and practical knowledge that any probationer trained under her is speedily snapped up when she may want a change of work. Miss Pountney is fortunate in having a committee who are genuinely interested in the progress of the hospital, and also a ladies' committee, who do a large amount of valuable work. They possess the merit of never interfering with the business of other people or of ever going outside their own allotted duties. Thirty years' continuous superintendence of a children's hospital, with ever-increasing success, constitutes a record, and entitles Miss Pountney to the most generous consideration in regard to a pension at the hands of her committee, whenever she may claim the retirement which is hers by right, at her discretion.

The hospital contains a unique recognition of the work of its founder, whose name is not perpetuated as it ought to be in the annual report, so far as our observation goes. This memorial consists of a panel of black oak carved by a grateful patient, which contains in the centre the founder's initials, below which the date of his death is recorded. We would suggest that this interesting and decorative memorial, which is as simple as it is effective and unique, should be framed so as to form the centre of a raised and ornamental frame, which should be closed by a piece of the best bevelled glass. If this were done it would prove a most striking addition to the board-room.

We have spoken in strong terms of the overcrowding of the large children's ward in one of the general hospitals at Sheffield. We are glad to note that overcrowding is forbidden in the Children's Hospital, the wards of which are airy, attractive, and good of their kind. The hospital has the merit of being able to show that each addition to its ward accommodation has been an improvement, and that the latest addition is the best of all—namely, the small verandas running the whole length of the ward, which are the best type possible in a climate and atmosphere like that of the city of Sheffield. This is a nice little Children's Hospital, containing sixty beds, with an average occupancy of fifty-four. It is well found and equipped with excellent accommodation for its nurses. In several respects, the latter being one, it sets an example to the managers of every other local hospital. The operation theatre is placed at the top of the hospital—why?—and as it is in direct communication by means of a large and, as we found it, open hatch with the smaller room adjacent, which contains a good-sized slop-sink, etc., it seems probable that the atmosphere of the operation-room may at times be adversely affected by it. There is a useful garden attached to the hospital, with a good-sized shelter for the use of the children when the weather permits.

### LXV.—Hospital for Sick Children, Newcastle-on-Tyne.

This hospital is housed in a relatively new building (1888), with an imposing elevation on Moor Edge, and its out-patients are provided for in an equally imposing block of buildings (Lady Armstrong Memorial), situated in the City Road. They afford an example of expensive hospital buildings, which it ought to have been possible to make more suitable for their purpose, than they are at present, by a smaller expenditure upon bricks and mortar.

We inspected both departments of the hospital, and formed the opinion in each case that what the hospital needs is some one person in authority, with a directing mind, who is well up to date in modern hospital administration. Such an administrator to succeed must be zealously earnest in enforcing efficiency and the prevention of old-fashioned hospital abuses, like overcrowding and the admission of many more patients than the accommodation, in the in-patient department, especially, warrants. If anybody is sufficiently interested to wish to understand exactly what we mean, we would suggest that they should visit the board-room provided at the in-patient department at Moor Edge, the plan of which, and its condition and contents, as we saw them after our inspections, graphically recalled and enforced the impression, which our visits had made, of the administrative shortcomings and their remedy.

The matron was out when we visited the in-patient department, but we were courteously received by the sister-in-charge, whose wards were in better order and better kept than any other portions of the hospital. The operation theatre more resembled a store than an aseptic up-to-date chamber, where many operations are performed each week. This habit of filling up space with various things, which are distinctly out of place where we saw them, seems to have been fallen into here, and ought speedily to be fallen out of. We have no doubt that good work is done by this hospital for the poor, but the quality of that work and the reputation and efficiency of everything connected with it, ought, in our judgment, speedily to attain to a much higher level than it has at present reached.

Had it not been for the cheery energy and evident interest and knowledge displayed by the lady who took us over the hospital, the feeling of depression with which we left it would have been grievous indeed. Both the out-patient and in-patient department of this hospital, in our view, require "dusting out" with continuous vigour, by some energetic and knowledgeable person who is heartily devoted to its interests and zealous for its reputation.

